# Supplementary material for: Changes in Children’s Adherence to Sustainable Healthy Diets During the Implementation of Chile’s Food Labelling and Advertising Law: A Longitudinal Study (2016–2019)
Source: Nutrients. 2025 Mar 16;17(6):1041. doi: 10.3390/nu17061041 (PMC11945912; doi:10.3390/nu17061041)
Supplement: Supplementary file 1 [file nutrients-17-01041-s001.zip › nutrients-3525397-supplementary.pdf]

Supplementary Materials

Article

Changes in Children's Adherence to Sustainable Healthy Diets During the Implementation of Chile's Food Labelling and Advertising Law: A Longitudinal Study (2016–2019)

Carolina Venegas Hargous [1], Liliana Orellana [2], Camila Corvalan [3], Steven Allender [1] and Colin Bell [1,4,*]

[1] Global Centre for Preventive Health and Nutrition (GLOBE), Institute for Health Transformation, Deakin University, Geelong 3220, Australia; c.venegashargous@deakin.edu.au (C.V.H.); steven.allender@deakin.edu.au (S.A.)
[2] Biostatistics Unit, Faculty of Health, Deakin University, Geelong 3220, Australia; l.orellana@deakin.edu.au
[3] Institute of Nutrition and Food Technology (INTA), University of Chile, Santiago 8330111, Chile; ccorvalan@inta.uchile.cl
[4] School of Medicine, Faculty of Health, Deakin University, Geelong 3220, Australia
* Correspondence: colin.bell@deakin.edu.au

Table of Contents

Table S1. STROBE-nut: An extension of the STROBE statement for nutritional epidemiology. 3

Figure S1. Participant flow chart........ 10

Table S2. Characteristics of participants included vs excluded from the analytical sample... 11

Table S3. Participant's characteristics at baseline (2016) and follow-up years (2017-2019)..... 13

Table S4. Non-adjusted changes in children's percentage of total caloric intake from the PHDI-C components after the first and second phases of the implementation of Chile's Food Labelling and Advertising Law (n=698). 15

Table S5. Non-adjusted changes in children's PHDI-C total and individual component scores after the first and second phases of the implementation of Chile's Food Labelling and Advertising Law (n=698)........ 17

Table S6. Adjusted changes in children's percentage of total caloric intake from the PHDI-C components after the first and second phases of the implementation of Chile's Food Labelling and Advertising Law (n=877). 18

Table S7. Adjusted changes in children's PHDI-C total and individual component scores after the first and second phases of the implementation of Chile's Food Labelling and Advertising Law (n=877)........ 20

References 21

Table S1. STROBE-nut: An extension of the STROBE statement for nutritional epidemiology.

Item	Item No	STROBE Recommendation	Extension for Nutritional Epidemiology Studies (STROBE-nut)	Page No
**Title and abstract**	1	(*a*) Indicate the study's design with a commonly used term in the title or the abstract.	nut-1. State the dietary/nutritional assessment method(s) used in the title, abstract, or keywords.	1
		(*b*) Provide in the abstract an informative and balanced summary of what was done and what was found.		1
**Introduction**				
Background/rationale	2	Explain the scientific background and rationale for the investigation being reported.		2
Objectives	3	State specific objectives, including any prespecified hypotheses.		2
**Methods**				
Study design	4	Present key elements of study design early in the paper.		3
Setting	5	Describe the setting, locations, and relevant dates, including periods of recruitment, exposure, follow-up, and data collection.	nut-5. Describe any characteristics of the study settings that might affect the dietary intake or nutritional status of the participants, if applicable.	3
Participants	6	(a) Cohort study—give the eligibility criteria and the sources and methods of selection of participants. Describe methods of follow-up.	nut-6. Report particular dietary, physiological, or nutritional characteristics that were	3

			considered when selecting the target population.	
		(*b*) Cohort study—for matched studies, give matching criteria and number of exposed and unexposed.		NA
Variables	7	Clearly define all outcomes, exposures, predictors, potential confounders, and effect modifiers. Give diagnostic criteria, if applicable	nut-7.1. Clearly define foods, food groups, nutrients, or other food components.	4-5
			nut-7.2. When using dietary patterns or indices, describe the methods to obtain them and their nutritional properties.	4-5
Data sources/ measurement	8	For each variable of interest, give sources of data and details of methods of assessment (measurement). Describe comparability of assessment methods if there is more than one group	nut-8.1. Describe the dietary assessment method(s), e.g.,portion size estimation, number of days and items recorded, how it was developed and administered, and how quality was assured. Report if and how supplement intake was assessed.	3-4
			nut-8.2. Describe and justify food-composition data used; explain the procedure to match food composition with consumption data; describe the use of conversion	3-4

			factors, if applicable.	
			nut-8.3. Describe the nutrient requirements, recommendations, or dietary guidelines and the evaluation approach used to compare intake with the dietary reference values, if applicable.	4-5
			nut-8.4. When using nutritional biomarkers, additionally use the STROBE-ME; report the type of biomarkers used and usefulness as dietary exposure markers.	NA
			nut-8.5. Describe the assessment of nondietary data (e.g., nutritional status and influencing factors) and timing of the assessment of these variables in relation to dietary assessment.	3
			nut-8.6. Report on the validity of the dietary or nutritional assessment methods and any internal or external validation used in the study, if applicable.	4, 15

Bias	9	Describe any efforts to address potential sources of bias	nut-9. Report how bias in dietary or nutritional assessment was addressed (e.g., misreporting, changes in habits as a result of being measured, data imputation from other sources).	4, 15
Study size	10	Explain how the study size was arrived at		6, Figure S1
Quantitative variables	11	Explain how quantitative variables were handled in the analyses. If applicable, describe which groupings were chosen and why	nut-11. Explain categorization of dietary/nutritional data (e.g., use of N-tiles and handling of non consumers) and the choice of reference category, if applicable.	6
Statistical methods	12	(*a*) Describe all statistical methods, including those used to control for confounding	nut-12.1. Describe any statistical method used to combine dietary or nutritional data, if applicable.	6
			nut-12.2. Describe and justify the method for energy adjustments, intake modeling, and use of weighting factors, if applicable	NA
			nut-12.3. Report any adjustments for measurement error (i.e., from a validity or calibration study).	NA

		(*b*) Describe any methods used to examine subgroups and interactions		NA
		(*c*) Explain how missing data were addressed		6
		(*d*) If applicable, explain how loss to follow-up was addressed		6
		(*e*) Describe any sensitivity analyses		6
**Results**				
Participants	13	(a) Report numbers of individuals at each stage of study—eg numbers potentially eligible, examined for eligibility, confirmed eligible, included in the study, completing follow-up, and analysed	nut-13. Report the number of individuals excluded on the basis of missing, incomplete, or implausible dietary and nutritional data.	Figure S1
		(b) Give reasons for non-participation at each stage		Figure S1
		(c) Consider use of a flow diagram		Figure S1
Descriptive data	14	(a) Give characteristics of study participants (eg demographic, clinical, social) and information on exposures and potential confounders	nut-14. Give the distribution of participant characteristics across the exposure variables, if applicable; specify if food consumption for the total population or consumers only was used to obtain results	6, Table 1
		(b) Indicate number of participants with missing data for each variable of interest		Table 1
		(c) Summarise follow-up time (eg, average and total amount)		Figure S1

Outcome data	15*	Report numbers of outcome events or summary measures over time		7-8
Main results	16	(*a*) Give unadjusted estimates and, if applicable, confounder-adjusted estimates and their precision (eg, 95% confidence interval). Make clear which confounders were adjusted for and why they were included	nut-16. Specify if nutrient intakes are reported with or without the inclusion of dietary supplement intake, if applicable.	8-9, Tables 2 & 3
		(*b*) Report category boundaries when continuous variables were categorized		Tables 1, 2 & 3
		(*c*) If relevant, consider translating estimates of relative risk into absolute risk for a meaningful time period.		NA
Other analyses	17	Report other analyses done—eg analyses of subgroups and interactions, and sensitivity analyses	nut-17. Report any sensitivity analysis (e.g., exclusion of misreporters or outliers) and data imputation, if applicable.	9, Tables S6 and 7
**Discussion**				
Key results	18	Summarise key results with reference to study objectives		14
Limitations	19	Discuss limitations of the study, taking into account sources of potential bias or imprecision. Discuss both direction and magnitude of any potential bias	nut-19. Describe the main limitations of the data sources and assessment methods used and implications for the interpretation of the findings.	16-17
Interpretation	20	Give a cautious overall interpretation of results considering objectives, limitations, multiplicity of analyses, results from similar	nut-20. Report the nutritional relevance of the findings, given the complexity of	14-16

		studies, and other relevant evidence	diet or nutrition as an exposure.	
Generalisability	21	Discuss the generalisability (external validity) of the study results		16-17
**Other information**				
Funding	22	Give the source of funding and the role of the funders for the present study and, if applicable, for the original study on which the present article is based		18
Ethics			nut-22.1. Describe the procedure for consent and study approval from ethics committee(s).	18
Supplementary Material			Nut-22.2. Provide data collection tools and data as online material or explain how they can be accessed.	17-18

Figure S1. Participant flow chart.

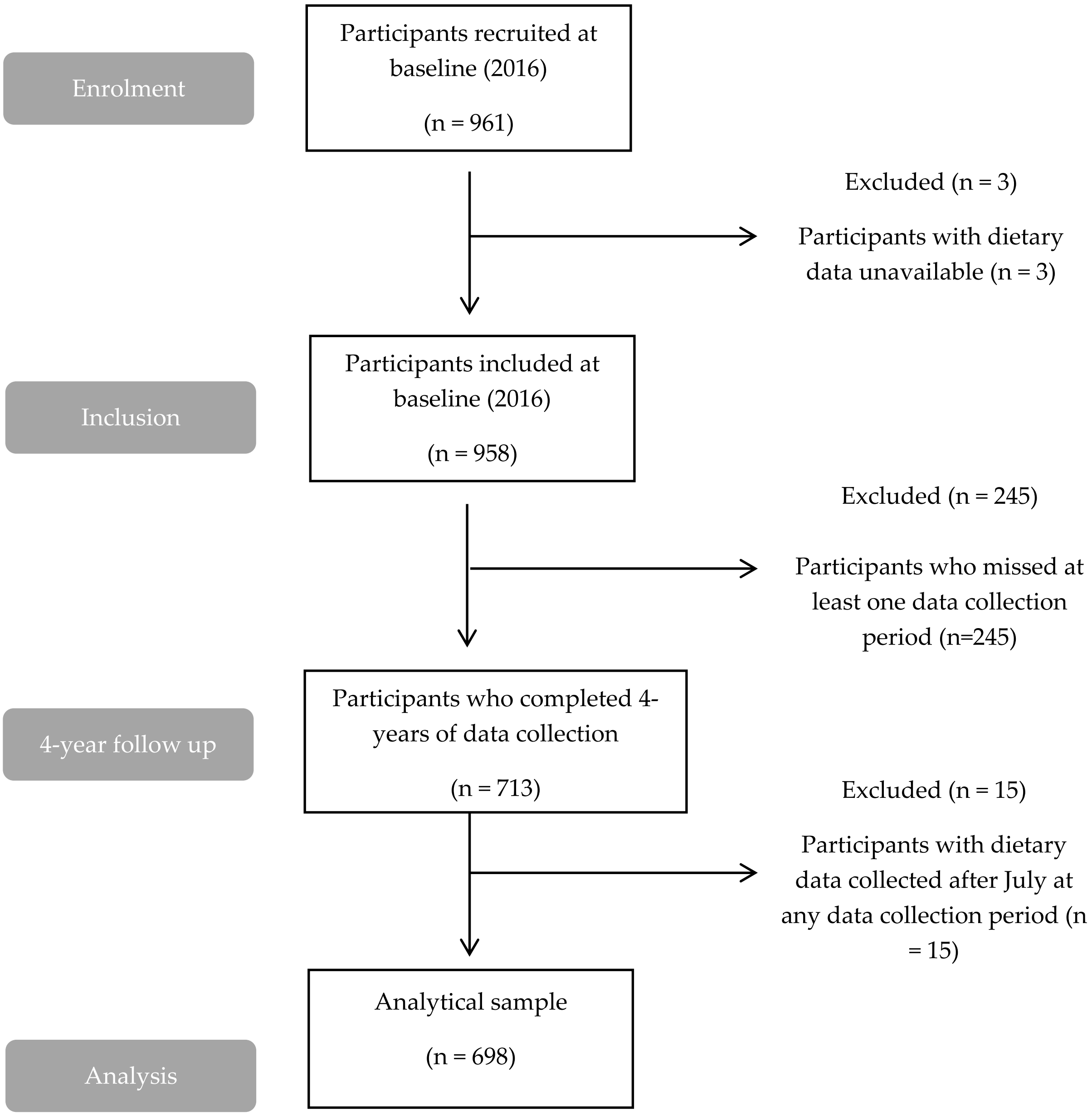

Table S2. Characteristics of participants included vs excluded from the analytical sample.

	Whole sample (n=958) n (%)	Analytical sample (n=698) n (%)	Participants excluded (n=260) n (%)	p-value [h]
**Sociodemographic characteristics**				
**Child gender**				
Male	462 (48.2)	351 (50.3)	111 (42.7)	0.036
Female	496 (51.8)	347 (49.7)	149 (57.3)	
**Child age**				
3-4 years	695 (72.6)	505 (72.4)	190 (73.1)	0.823
5-6 years	263 (77.4)	193 (27.6)	70 (26.9)	
**Maternal age**				
< 25 years	167 (17.4)	123 (17.6)	44 (16.9)	0.800
≥ 25 years	791 (82.6)	575 (82.4)	216 (83.1)	
**Maternal level of education**				
Incomplete secondary education	173 (18.1)	108 (15.4)	65 (25.0)	0.002
Complete secondary education	529 (55.2)	404 (57.9)	125 (48.1)	
Complete tertiary education	256 (26.7)	186 (26.7)	70 (26.9)	
**Anthropometric characteristics**				
**Child weight status** [a]				
At risk of undernutrition	23 (2.4)	21 (3.0)	2 (0.8)	0.065
Normal weight	483 (50.4)	357 (51.2)	126 (48.7)	
Overweight	275 (28.7)	204 (29.2)	71 (27.4)	
Obesity	118 (12.3)	77 (11.0)	41 (15.8)	
Severe obesity	58 (6.1)	39 (5.6)	19 (7.3)	
**Maternal weight status** [b,c]				
Underweight	5 (0.5)	5 (0.8)	0 (0.0)	0.192
Normal weight	241 (25.1)	188 (28.1)	53 (21.3)	
Overweight	345 (36.0)	241 (36.0)	104 (41.8)	
Obesity class I	217 (22.7)	155 (23.2)	62 (24.9)	
Obesity class II	75 (7.8)	53 (7.9)	22 (8.8)	
Obesity class III	35 (3.7)	27 (4.0)	8 (3.2)	
**Maternal abdominal obesity** [c,d]				
Absence	408 (42.6)	303 (45.3)	105 (42.2)	0.397
Presence	510 (53.2)	366 (54.7)	144 (57.8)	
**Dietary recall characteristics**				
**Day of the dietary recall**				
Weekday	821 (85.7)	598 (85.7)	37 (14.2)	0.970
Weekend day/holiday	137 (14.3)	100 (14.3)	223 (85.8)	
**Type of eating pattern** [e]				
Typical	801 (83.6)	586 (84.0)	215 (82.7)	0.639
Atypical	157 (16.4)	112 (16.0)	45 (17.3)	

**Type of diet** [f]				
Normal	905 (94.5)	661 (94.7)	244 (93.9)	0.608
Special	53 (5.5)	37 (5.3)	16 (6.1)	
**Reliability of the dietary recall** [g]				
Reliable	904 (94.4)	654 (93.7)	250 (96.2)	0.142
Unreliable	54 (5.6)	44 (6.3)	10 (3.85)	

[a] Child weight status was defined according to WHO Child Growth Standards 2006 [1] for children under 5-years-old, and WHO Growth Reference 2007 [2] for children above 5-years of age.

[b] Maternal weight status was defined using the WHO cut-off points for BMI in adults [3].

[c] Missing data for 29 participants included in analytical sample (28 mothers were pregnant and 1 refused to be measured); Missing data for 11 participants excluded from analytical sample (9 mothers were pregnant and 2 refused to be measured).

[d] Presence of abdominal obesity in children's mothers was defined using the Adult Treatment Panel III criteria for clinical identification of metabolic syndrome (waist circumference above 88 cm) [4].

[e] Typical eating pattern refers to a recall from a regular day; atypical eating pattern refers to a recall from a special occasion such celebration, vacation, or sickness.

[f] Normal diet refers to an omnivorous diet with no dietary restriction of any kind; special diet refers to lactose free, gluten free, vegetarian, or vegan diets.

[g] Unreliable recalls refer to recalls where there was missing information on the amount consumed of some food items.

[h] P-values obtained using the chi-square test.

Table S3. Participant's characteristics at baseline (2016) and follow-up years (2017-2019).

	2016	2017	2018	2019
**Sociodemographic characteristics**				
**Child age, mean (SD)**	4.3 (0.5)	5.2 (0.5)	6.1 (0.5)	7.1 (0.5)
**Maternal age, mean (SD)**	31.5 (6.6)	32.4 (6.6)	33.3 (6.6)	34.2 (6.6)
**Maternal level of education, n (%)**				
Incomplete secondary education	108 (15.4)	103 (14.8)	103 (14.8)	103 (14.8)
Complete secondary education	404 (57.9)	392 (56.2)	391 (56.0)	378 (54.1)
Complete tertiary education	186 (26.7)	203 (29.1)	204 (29.2)	217 (31.1)
**Anthropometric characteristics**				
**Child weight status, n (%)** [a]				
At risk of undernutrition	21 (3.0)	18 (2.6)	30 (4.3)	22 (3.2)
Normal weight	357 (51.2)	357 (51.4)	319 (45.8)	295 (42.5)
Overweight	204 (29.2)	203 (29.2)	200 (28.7)	202 (29.1)
Obesity	77 (11.0)	76 (10.9)	106 (15.2)	122 (17.6)
Severe obesity	39 (5.6)	41 (5.9)	41 (5.9)	53 (7.6)
**Maternal weight status, n (%)** [b,c]				
Underweight	5 (0.8)	2 (0.3)	1 (0.2)	3 (0.5)
Normal weight	188 (28.1)	174 (25.6)	153 (22.7)	150 (22.4)
Overweight	241 (36.0)	266 (39.2)	262 (38.9)	255 (38.1)
Obesity class I	155 (23.2)	151 (22.2)	165 (24.5)	166 (24.8)
Obesity class II	53 (7.9)	59 (8.7)	64 (9.5)	67 (10.0)
Obesity class III	27 (4.0)	27 (4.0)	29 (4.3)	28 (4.2)
**Maternal abdominal obesity, n (%)** [c,d]				
Absence	303 (45.3)	288 (42.8)	266 (39.6)	265 (39.7)
Presence	366 (54.7)	385 (57.2)	406 (60.4)	402 (60.3)
**Dietary recall characteristics**				
**Day of the dietary recall, n (%)**				
Weekday	598 (85.7)	511 (73.2)	595 (85.2)	589 (84.4)
Weekend day/holiday	100 (14.3)	187 (26.8)	103 (14.8)	109 (15.6)
**Type of eating pattern, n (%)** [e]				
Typical	586 (84.0)	578 (82.8)	626 (89.7)	575 (82.4)
Atypical	112 (16.0)	120 (17.2)	72 (10.3)	123 (17.6)
**Type of diet, n (%)** [f]				
Normal	661 (94.7)	673 (96.4)	669 (95.9)	661 (94.7)
Special	37 (5.3)	25 (3.6)	29 (4.1)	37 (5.3)
**Reliability of the dietary recall, n (%)** [g]				
Reliable	654 (93.7)	683 (97.9)	665 (95.3)	669 (95.9)
Unreliable	44 (6.3)	15 (2.1)	33 (4.7)	29 (4.1)
**Diet nutritional composition, mean (SD)**				
Energy				
Total energy intake, kcal/day	1181.4 (376.8)	1316.8 (413.6)	1309.6 (391.4)	1464.6 (581.1)
**Macronutrients**				
Total protein intake, % total energy	14.1 (3.8)	14.6 (3.7)	14.2 (3.7)	14.3 (3.9)
Animal-based protein intake, % total energy	9.8 (3.9)	10.0 (4.0)	9.6 (4.0)	9.8 (4.0)

Plant-based proteins intake, % total energy	4.3 (2.6)	4.5 (2.1)	4.6 (2.1)	4.6 (2.0)
Total fats intake, % total energy	28.8 (6.4)	30.1 (7.0)	30.4 (7.0)	30.8 (7.2)
Saturated fats intake, % total energy	10.4 (3.4)	11.0 (3.7)	11.1 (3.6)	11.1 (4.0)
Trans fats intake, % total energy	0.5 (0.3)	0.6 (0.4)	0.5 (0.6)	0.5 (0.5)
Total carbohydrates intake, % total energy	57.8 (7.6)	56.2 (7.6)	53.6 (10.9)	53.1 (11.3)
Total sugars intake, % total energy	28.9 (8.8)	24.6 (7.4)	24.1 (7.3)	23.7 (8.3)
Total fibre intake, g/1000kcal	7.2 (4.4)	7.6 (4.5)	9.3 (4.5)	9.4 (4.2)

[a] Child weight status was defined according to WHO Child Growth Standards 2006 [1] for children under 5-years-old, and WHO Growth Reference 2007 [2] for children above 5-years of age.

[b] Maternal weight status was defined using the WHO cut-off points for BMI in adults [3].

[c] Missing data for 29 participants; 28 mothers were pregnant and 1 refused to be measured.

[d] Presence of abdominal obesity in children's mothers was defined using the Adult Treatment Panel III criteria for clinical identification of metabolic syndrome (waist circumference above 88 cm) [4].

[e] Typical eating pattern refers to a recall from a regular day; atypical eating pattern refers to a recall from a special occasion such celebration, vacation, or sickness.

[f] Normal diet refers to an omnivorous diet with no dietary restriction of any kind; special diet refers to lactose free, gluten free, vegetarian, or vegan diets.

[g] Unreliable recalls refer to recalls where there was missing information on the amount consumed of some food items.

Table S4. Non-adjusted changes in children's percentage of total caloric intake from the PHDI-C components after the first and second phases of the implementation of Chile's Food Labelling and Advertising Law (n=698).

PHDI-C components	PHDI-C recommended percentage of total caloric intake for children [a]	Children's percentage of total caloric intake [a, b]									
		2016	2017	2018	2019	2017 vs 2016		2018 vs 2016		2019 vs 2016	
	Optimal value (range)	mean (95%CI)	mean (95%CI)	mean (95%CI)	mean (95%CI)	diff (95%CI)	p-value	diff (95%CI)	p-value	diff (95%CI)	p-value
**Adequacy components**											
Nuts & peanuts	≥ 11.6 (0, 100)	0.34 (0.19, 0.49)	0.39 (0.24, 0.54)	0.43 (0.28, 0.58)	0.27 (0.12, 0.42)	0.05 (-0.16, 0.25)	0.641	0.09 (-0.11, 0.3)	0.381	-0.07 (-0.28, 0.13)	0.499
Legumes	≥ 11.3 (0, 100)	2.27 (1.92, 2.61)	1.92 (1.57, 2.26)	1.65 (1.3, 1.99)	1.06 (0.72, 1.41)	-0.35 (-0.83, 0.13)	0.152	-0.62 (-1.1, -0.14)	0.011	-1.21 (-1.68, -0.73)	<0.001
Fruits	≥ 5.0 (0, 100)	4.97 (4.58, 5.37)	4.67 (4.28, 5.06)	4.57 (4.18, 4.97)	4.5 (4.11, 4.9)	-0.3 (-0.81, 0.2)	0.241	-0.4 (-0.9, 0.1)	0.120	-0.47 (-0.97, 0.03)	0.068
Vegetables	≥ 3.1 (0, 100)	1.43 (1.32, 1.54)	1.45 (1.34, 1.57)	1.3 (1.19, 1.41)	1.48 (1.36, 1.59)	0.02 (-0.12, 0.17)	0.745	-0.13 (-0.27, 0.01)	0.074	0.05 (-0.1, 0.19)	0.539
**Ratio components**											
DGV ratio	29.5 (0, 100)	5.58 (4.32, 6.84)	5.13 (3.87, 6.39)	4.63 (3.37, 5.89)	4.42 (3.16, 5.68)	-0.45 (-2.19, 1.3)	0.615	-0.95 (-2.7, 0.79)	0.284	-1.16 (-2.9, 0.59)	0.193
ReV ratio	38.5 (0, 100)	40.39 (38, 42.78)	39.47 (37.07, 41.86)	36.87 (34.48, 39.27)	38.67 (36.28, 41.06)	-0.93 (-4.18, 2.32)	0.576	-3.52 (-6.77, -0.27)	0.034	-1.72 (-4.97, 1.53)	0.300
WC ratio	75.0 (0, 100)	4.85 (3.69, 6)	7.66 (6.5, 8.81)	6.25 (5.09, 7.4)	9.24 (8.09, 10.4)	2.81 (1.28, 4.35)	<0.001	1.4 (-0.14, 2.94)	0.074	4.4 (2.86, 5.94)	<0.001
**Optimum components**											
Cereals	30.0 (0, 60.0)	25.4 (24.64, 26.16)	26.86 (26.09, 27.62)	28.82 (28.06, 29.58)	29.47 (28.70, 30.23)	1.46 (0.44, 2.47)	0.005	3.42 (2.41, 4.44)	<0.001	4.07 (3.05, 5.08)	<0.001
Tubers & potatoes	1.6 (0, 3.1)	3.38 (2.98, 3.78)	3.78 (3.38, 4.17)	3.67 (3.28, 4.07)	3.68 (3.28, 4.08)	0.4 (-0.16, 0.95)	0.163	0.29 (-0.26, 0.85)	0.302	0.30 (-0.26, 0.85)	0.292
Dairy products	12.2 (0, 24.4)	19.96 (19.18, 20.75)	18.62 (17.83, 19.4)	16.65 (15.86, 17.44)	16.03 (15.24, 16.81)	-1.35 (-2.3, -0.39)	0.006	-3.31 (-4.27, -2.36)	<0.001	-3.94 (-4.89, -2.98)	<0.001
Eggs & white meats	6.2 (0, 12.2)	5.7 (5.16, 6.25)	6.11 (5.56, 6.65)	6.59 (6.04, 7.13)	6.58 (6.04, 7.13)	0.41 (-0.33, 1.14)	0.279	0.89 (0.15, 1.62)	0.019	0.88 (0.15, 1.61)	0.019
Vegetable oils	14.1 (0, 28.3)	10.4 (9.88, 10.91)	10.36 (9.85, 10.87)	9.78 (9.28, 10.29)	10.00 (9.49, 10.51)	-0.04 (-0.72, 0.65)	0.914	-0.61 (-1.3, 0.07)	0.079	-0.4 (-1.09, 0.28)	0.252
**Moderation components**											
Palm oil	0.0 (0, 2.4)	3.41 (3.06, 3.76)	3.69 (3.34, 4.05)	4.51 (4.16, 4.87)	4.7 (4.35, 5.05)	0.28 (-0.18, 0.75)	0.234	1.11 (0.64, 1.58)	<0.001	1.29 (0.82, 1.76)	<0.001
Red meats	0.0 (0, 2.4)	4.39 (3.85, 4.93)	5.48 (4.94, 6.02)	5.68 (5.14, 6.22)	5.62 (5.08, 6.16)	1.09 (0.36, 1.82)	0.004	1.29 (0.56, 2.02)	0.001	1.23 (0.50, 1.96)	0.001
Animal fats	0.0 (0, 1.4)	1.75 (1.51, 1.99)	2.31 (2.07, 2.55)	2.34 (2.1, 2.58)	2.41 (2.17, 2.65)	0.56 (0.23, 0.89)	0.001	0.59 (0.26, 0.93)	0.001	0.66 (0.32, 1.00)	<0.001
Added sugars	0.0 (0, 4.8)	16.24 (15.66, 16.82)	13.7 (13.12, 14.28)	13.68 (13.1, 14.26)	13.93 (13.35, 14.51)	-2.54 (-3.29, -1.8)	<0.001	-2.56 (-3.31, -1.81)	<0.001	-2.31 (-3.06, -1.56)	<0.001

Abbreviations: PHDI-C, Planetary Health Diet Index for children and adolescents; DGV ratio, dark green vegetables ratio; ReV ratio, red and orange vegetables ratio; WC ratio, whole cereals ratio; CI, confidence interval; diff, difference.

[a] Values are expressed as percentage of total caloric intake, except for the DGV ratio & ReV ratio components where values are expressed as percentage of total calories from vegetables, and for the WC ratio component where values are expressed as percentage of total calories from cereals.

[b] Estimates and p-values from non-adjusted mixed effects models; n=698.

Table S5. Non-adjusted changes in children's PHDI-C total and individual component scores after the first and second phases of the implementation of Chile's Food Labelling and Advertising Law (n=698).

PHDI-C components	PHDI-C possible scores	Participants' PHDI-C scores [a]									
		2016	2017	2018	2019	2017 vs 2016		2018 vs 2016		2019 vs 2016	
	Points	mean (95%CI)	mean (95%CI)	mean (95%CI)	mean (95%CI)	diff (95%CI)	p-value	diff (95%CI)	p-value	diff (95%CI)	p-value
**Adequacy components**											
Nuts & peanuts	0 – 10	0.23 (0.13, 0.32)	0.29 (0.2, 0.39)	0.33 (0.24, 0.43)	0.21 (0.11, 0.31)	0.07 (-0.07, 0.2)	0.317	0.11 (-0.03, 0.24)	0.117	-0.01 (-0.15, 0.12)	0.859
Legumes	0 – 10	1.48 (1.27, 1.69)	1.33 (1.12, 1.54)	1.15 (0.94, 1.36)	0.75 (0.54, 0.97)	-0.15 (-0.44, 0.14)	0.317	-0.34 (-0.63, -0.04)	0.024	-0.73 (-1.02, -0.44)	<0.001
Fruits	0 – 10	6.02 (5.71, 6.33)	5.57 (5.26, 5.88)	5.67 (5.36, 5.99)	5.32 (5.01, 5.63)	-0.45 (-0.85, -0.05)	0.026	-0.35 (-0.74, 0.05)	0.087	-0.7 (-1.1, -0.3)	0.001
Vegetables	0 – 10	4.2 (3.96, 4.45)	4.04 (3.79, 4.28)	3.74 (3.5, 3.99)	4.10 (3.85, 4.34)	-0.17 (-0.48, 0.14)	0.293	-0.46 (-0.77, -0.15)	0.004	-0.11 (-0.42, 0.2)	0.502
**Ratio components**											
DGV ratio	0 – 5	0.36 (0.29, 0.44)	0.34 (0.27, 0.41)	0.24 (0.16, 0.31)	0.25 (0.18, 0.32)	-0.02 (-0.12, 0.08)	0.655	-0.13 (-0.23, -0.03)	0.011	-0.12 (-0.21, -0.02)	0.021
ReV ratio	0 – 5	2.38 (2.24, 2.51)	2.14 (2.01, 2.28)	1.93 (1.79, 2.06)	2.06 (1.93, 2.2)	-0.23 (-0.42, -0.05)	0.014	-0.45 (-0.64, -0.26)	<0.001	-0.31 (-0.5, -0.13)	0.001
WC ratio	0 – 10	0.58 (0.45, 0.72)	0.86 (0.73, 1.00)	0.81 (0.68, 0.94)	1.10 (0.97, 1.23)	0.28 (0.11, 0.45)	0.002	0.23 (0.05, 0.40)	0.010	0.51 (0.34, 0.69)	<0.001
**Optimum components**											
Cereals	0 – 10	7.06 (6.9, 7.22)	7.2 (7.04, 7.36)	7.17 (7.02, 7.33)	7.19 (7.03, 7.35)	0.14 (-0.08, 0.36)	0.218	0.11 (-0.11, 0.33)	0.319	0.13 (-0.09, 0.35)	0.262
Tubers & potatoes	0 – 10	0.47 (0.36, 0.58)	0.3 (0.19, 0.41)	0.36 (0.24, 0.47)	0.32 (0.21, 0.43)	-0.17 (-0.32, -0.01)	0.035	-0.11 (-0.27, 0.04)	0.161	-0.14 (-0.3, 0.01)	0.073
Dairy products	0 – 10	3.78 (3.52, 4.04)	4.12 (3.87, 4.38)	4.29 (4.04, 4.55)	4.24 (3.99, 4.50)	0.34 (-0.00, 0.68)	0.052	0.51 (0.17, 0.85)	0.004	0.46 (0.12, 0.81)	0.008
Eggs & white meats	0 – 10	2.87 (2.62, 3.13)	2.92 (2.66, 3.17)	3.00 (2.74, 3.26)	2.96 (2.71, 3.22)	0.04 (-0.32, 0.4)	0.811	0.13 (-0.24, 0.49)	0.495	0.09 (-0.27, 0.45)	0.628
Vegetable oils	0 – 10	5.57 (5.36, 5.78)	5.33 (5.12, 5.54)	5.17 (4.96, 5.38)	5.01 (4.80, 5.22)	-0.24 (-0.52, 0.05)	0.105	-0.4 (-0.69, -0.12)	0.006	-0.56 (-0.85, -0.27)	<0.001
**Moderation components**											
Palm oil	0 – 10	4.73 (4.4, 5.06)	4.31 (3.98, 4.64)	3.61 (3.28, 3.94)	3.46 (3.12, 3.79)	-0.42 (-0.87, 0.03)	0.066	-1.11 (-1.56, -0.67)	<0.001	-1.27 (-1.71, -0.82)	<0.001
Red meats	0 – 10	4.9 (4.54, 5.26)	4.4 (4.05, 4.76)	4.25 (3.89, 4.61)	4.15 (3.79, 4.51)	-0.49 (-0.99, 0)	0.05	-0.65 (-1.14, -0.16)	0.01	-0.75 (-1.24, -0.25)	0.003
Animal fats	0 – 10	5.41 (5.05, 5.76)	4.62 (4.27, 4.97)	4.53 (4.17, 4.88)	4.41 (4.06, 4.77)	-0.79 (-1.27, -0.3)	0.001	-0.88 (-1.36, -0.39)	<0.001	-0.99 (-1.48, -0.51)	<0.001
Added sugars	0 – 10	0.17 (0.06, 0.28)	0.42 (0.31, 0.53)	0.41 (0.3, 0.52)	0.49 (0.38, 0.60)	0.25 (0.09, 0.4)	0.001	0.24 (0.09, 0.39)	0.002	0.31 (0.16, 0.47)	<0.001
**Total PHDI-C score**	0 - 150	50.22 (49.16, 51.27)	48.21 (47.15, 49.26)	46.66 (45.61, 47.72)	46.03 (44.98, 47.08)	-2.01 (-3.37, -0.65)	0.004	-3.55 (-4.92, -2.19)	<0.001	-4.19 (-5.55, -2.82)	<0.001

Abbreviations: PHDI-C, Planetary Health Diet Index for children and adolescents; DGV ratio, dark green vegetables ratio; ReV ratio, red and orange vegetables ratio; WC ratio, whole cereals ratio; CI, confidence interval; diff, difference.

[a] Estimates and p-values from non-adjusted mixed effects models; n=698.

Table S6. Adjusted changes in children's percentage of total caloric intake from the PHDI-C components after the first and second phases of the implementation of Chile's Food Labelling and Advertising Law (n=877).

PHDI-C components	PHDI-C recommended percentage of total caloric intake for children [a]	Children's percentage of total caloric intake [a,b]									
		2016	2017	2018	2019	2017 vs 2016		2018 vs 2016		2019 vs 2016	
	Optimal value (range)	mean (95%CI)	mean (95%CI)	mean (95%CI)	mean (95%CI)	diff (95%CI)	p-value	diff (95%CI)	p-value	diff (95%CI)	p-value
**Adequacy components**											
Nuts & peanuts	≥ 11.6 (0, 100)	0.31 (0.17, 0.45)	0.4 (0.26, 0.54)	0.45 (0.3, 0.59)	0.31 (0.16, 0.46)	0.09 (-0.1, 0.28)	0.367	0.14 (-0.06, 0.33)	0.162	0 (-0.2, 0.19)	0.982
Legumes	≥ 11.3 (0, 100)	2.33 (2.01, 2.65)	2.11 (1.78, 2.43)	1.6 (1.26, 1.94)	1.1 (0.75, 1.44)	-0.22 (-0.67, 0.23)	0.337	-0.73 (-1.19, -0.27)	0.002	-1.23 (-1.69, -0.77)	<0.001
Fruits	≥ 5.0 (0, 100)	5.01 (4.66, 5.36)	4.96 (4.61, 5.31)	4.57 (4.2, 4.94)	4.51 (4.14, 4.89)	-0.05 (-0.5, 0.4)	0.828	-0.44 (-0.9, 0.02)	0.062	-0.5 (-0.97, -0.03)	0.037
Vegetables	≥ 3.1 (0, 100)	1.46 (1.36, 1.57)	1.51 (1.41, 1.61)	1.3 (1.19, 1.41)	1.51 (1.39, 1.62)	0.04 (-0.09, 0.18)	0.517	-0.17 (-0.3, -0.03)	0.019	0.04 (-0.1, 0.18)	0.573
**Ratio components**											
DGV ratio	29.5 (0, 100)	5.41 (4.29, 6.53)	4.87 (3.73, 6.01)	4.68 (3.49, 5.87)	4.38 (3.17, 5.6)	-0.54 (-2.11, 1.03)	0.503	-0.73 (-2.33, 0.87)	0.374	-1.03 (-2.64, 0.59)	0.214
ReV ratio	38.5 (0, 100)	39.72 (37.59, 41.86)	40.7 (38.53, 42.87)	36.38 (34.11, 38.64)	39.24 (36.93, 41.55)	0.97 (-1.97, 3.92)	0.517	-3.35 (-6.35, -0.34)	0.029	-0.49 (-3.52, 2.55)	0.754
WC ratio	75.0 (0, 100)	4.89 (3.88, 5.9)	7.47 (6.44, 8.5)	6.01 (4.93, 7.08)	9.35 (8.25, 10.44)	2.58 (1.21, 3.96)	<0.001	1.12 (-0.28, 2.52)	0.117	4.46 (3.05, 5.88)	<0.001
**Optimum components**											
Cereals	30.0 (0, 60.0)	24.97 (24.3, 25.65)	26.87 (26.18, 27.55)	28.81 (28.1, 29.53)	29.44 (28.71, 30.17)	1.89 (0.98, 2.81)	<0.001	3.84 (2.9, 4.77)	<0.001	4.46 (3.52, 5.41)	<0.001
Tubers & potatoes	1.6 (0, 3.1)	3.46 (3.11, 3.81)	3.75 (3.39, 4.1)	3.59 (3.22, 3.97)	3.69 (3.31, 4.07)	0.29 (-0.21, 0.79)	0.25	0.14 (-0.37, 0.64)	0.591	0.23 (-0.28, 0.74)	0.372
Dairy products	12.2 (0, 24.4)	20.04 (19.35, 20.74)	18.85 (18.14, 19.55)	16.65 (15.92, 17.38)	16 (15.26, 16.75)	-1.2 (-2.06, -0.33)	0.007	-3.39 (-4.28, -2.51)	<0.001	-4.04 (-4.93, -3.15)	<0.001
Eggs & white meats	6.2 (0, 12.2)	5.87 (5.38, 6.36)	6.25 (5.75, 6.74)	6.5 (5.99, 7.02)	6.54 (6.02, 7.07)	0.38 (-0.29, 1.04)	0.267	0.63 (-0.05, 1.31)	0.068	0.67 (-0.02, 1.36)	0.055
Vegetable oils	14.1 (0, 28.3)	10.29 (9.84, 10.74)	10.27 (9.81, 10.73)	9.84 (9.36, 10.31)	10.1 (9.62, 10.59)	-0.02 (-0.63, 0.6)	0.96	-0.45 (-1.08, 0.18)	0.161	-0.18 (-0.82, 0.45)	0.571
**Moderation components**											
Palm oil	0.0 (0, 2.4)	3.41 (3.1, 3.72)	3.67 (3.35, 3.98)	4.43 (4.1, 4.76)	4.72 (4.38, 5.05)	0.26 (-0.16, 0.68)	0.232	1.02 (0.59, 1.45)	<0.001	1.31 (0.87, 1.74)	<0.001
Red meats	0.0 (0, 2.4)	4.33 (3.85, 4.8)	5.37 (4.89, 5.86)	5.7 (5.19, 6.2)	5.65 (5.13, 6.16)	1.05 (0.4, 1.7)	0.002	1.37 (0.71, 2.03)	<0.001	1.32 (0.65, 1.99)	<0.001
Animal fats	0.0 (0, 1.4)	1.78 (1.56, 1.99)	2.16 (1.94, 2.37)	2.43 (2.2, 2.65)	2.4 (2.17, 2.63)	0.38 (0.09, 0.68)	0.011	0.65 (0.35, 0.95)	<0.001	0.63 (0.32, 0.93)	<0.001
Added sugars	0.0 (0, 4.8)	16.38 (15.87, 16.88)	13.21 (12.7, 13.72)	13.82 (13.29, 14.36)	13.78 (13.23, 14.32)	-3.17 (-3.83, -2.5)	<0.001	-2.55 (-3.24, -1.87)	<0.001	-2.6 (-3.29, -1.91)	<0.001

Abbreviations: PHDI-C, Planetary Health Diet Index for children and adolescents; DGV ratio, dark green vegetables ratio; ReV ratio, red and orange vegetables ratio; WC ratio, whole cereals ratio; CI, confidence interval; diff, difference.

a Values are expressed as percentage of total caloric intake, except for the DGV ratio & ReV ratio components where values are expressed as percentage of total calories from vegetables, and for the WC ratio component where values are expressed as percentage of total calories from cereals.

b Estimates and p-values from mixed effects models adjusting for dietary recall characteristics including: day of the dietary recall (weekday vs weekend/holiday), type of eating pattern (typical (i.e., recall from a typical day) vs atypical (i.e., recall from a special occasion such as celebrations, sickness or vacations)), and type of diet (normal (i.e., omnivorous diet with no dietary restriction of any kind) vs special diet (e.g., lactose free, gluten free, vegan or vegetarian diets)), plus child and maternal characteristics at baseline, including: child gender (male vs female), child age (3-4 years vs 5-6 years), child weight status (non-overweight, overweight, and obesity), maternal age (<25 years vs ≥25 years), and maternal education (incomplete secondary education, complete secondary education, complete tertiary education); n=877 (considers participants who had data collected at baseline (2016) and at least one more wave was performed).

Table S7. Adjusted changes in children's PHDI-C total and individual component scores after the first and second phases of the implementation of Chile's Food Labelling and Advertising Law (n=877).

PHDI-C components	PHDI-C possible scores	Participants' PHDI-C scores [a]									
		2016	2017	2018	2019	2017 vs 2016		2018 vs 2016		2019 vs 2016	
	Points	mean (95%CI)	mean (95%CI)	mean (95%CI)	mean (95%CI)	diff (95%CI)	p-value	diff (95%CI)	p-value	diff (95%CI)	p-value
**Adequacy components**											
Nuts & peanuts	0 – 10	0.21 (0.12, 0.29)	0.3 (0.21, 0.4)	0.34 (0.24, 0.43)	0.24 (0.15, 0.34)	0.1 (-0.03, 0.22)	0.12	0.13 (0, 0.26)	0.044	0.04 (-0.09, 0.17)	0.568
Legumes	0 – 10	1.48 (1.29, 1.67)	1.42 (1.23, 1.61)	1.11 (0.91, 1.31)	0.77 (0.57, 0.98)	-0.06 (-0.32, 0.21)	0.682	-0.37 (-0.64, -0.1)	0.008	-0.71 (-0.98, -0.43)	<0.001
Fruits	0 – 10	5.98 (5.71, 6.25)	5.8 (5.52, 6.08)	5.64 (5.35, 5.92)	5.34 (5.05, 5.63)	-0.18 (-0.53, 0.18)	0.324	-0.34 (-0.71, 0.02)	0.064	-0.64 (-1.01, -0.27)	0.001
Vegetables	0 – 10	4.17 (3.95, 4.38)	4.18 (3.96, 4.4)	3.75 (3.52, 3.98)	4.14 (3.91, 4.38)	0.01 (-0.27, 0.3)	0.927	-0.42 (-0.71, -0.13)	0.005	-0.02 (-0.31, 0.27)	0.888
**Ratio components**											
DGV ratio	0 – 5	0.35 (0.29, 0.41)	0.32 (0.25, 0.38)	0.23 (0.16, 0.29)	0.24 (0.17, 0.31)	-0.03 (-0.12, 0.06)	0.495	-0.12 (-0.21, -0.03)	0.008	-0.11 (-0.2, -0.02)	0.017
ReV ratio	0 – 5	2.36 (2.24, 2.48)	2.18 (2.06, 2.3)	1.88 (1.75, 2)	2.05 (1.92, 2.18)	-0.17 (-0.34, -0.01)	0.041	-0.48 (-0.65, -0.31)	<0.001	-0.3 (-0.48, -0.13)	0.001
WC ratio	0 – 10	0.6 (0.49, 0.72)	0.85 (0.73, 0.96)	0.78 (0.66, 0.9)	1.1 (0.98, 1.23)	0.24 (0.09, 0.4)	0.002	0.18 (0.02, 0.34)	0.025	0.5 (0.34, 0.66)	<0.001
**Optimum components**											
Cereals	0 – 10	7.02 (6.88, 7.16)	7.14 (7, 7.28)	7.18 (7.03, 7.33)	7.23 (7.08, 7.38)	0.11 (-0.08, 0.31)	0.259	0.16 (-0.05, 0.36)	0.133	0.21 (0, 0.41)	0.046
Tubers & potatoes	0 – 10	0.5 (0.4, 0.61)	0.32 (0.22, 0.42)	0.35 (0.24, 0.45)	0.32 (0.21, 0.43)	-0.18 (-0.33, -0.04)	0.013	-0.16 (-0.31, -0.01)	0.035	-0.19 (-0.34, -0.04)	0.013
Dairy products	0 – 10	3.75 (3.52, 3.98)	4.12 (3.89, 4.35)	4.23 (3.99, 4.47)	4.25 (4, 4.49)	0.37 (0.06, 0.68)	0.02	0.48 (0.16, 0.79)	0.003	0.5 (0.18, 0.82)	0.002
Eggs & white meats	0 – 10	2.85 (2.63, 3.08)	2.85 (2.62, 3.08)	2.96 (2.71, 3.2)	2.96 (2.72, 3.21)	0 (-0.33, 0.32)	0.979	0.1 (-0.23, 0.43)	0.545	0.11 (-0.22, 0.44)	0.519
Vegetable oils	0 – 10	5.57 (5.38, 5.75)	5.32 (5.13, 5.51)	5.18 (4.98, 5.38)	5.01 (4.8, 5.21)	-0.25 (-0.51, 0.01)	0.064	-0.38 (-0.65, -0.12)	0.005	-0.56 (-0.83, -0.29)	<0.001
**Moderation components**											
Palm oil	0 – 10	4.75 (4.46, 5.05)	4.35 (4.05, 4.65)	3.66 (3.35, 3.97)	3.47 (3.15, 3.79)	-0.4 (-0.8, 0)	0.051	-1.09 (-1.5, -0.69)	<0.001	-1.28 (-1.7, -0.87)	<0.001
Red meats	0 – 10	4.93 (4.61, 5.25)	4.43 (4.1, 4.75)	4.21 (3.87, 4.55)	4.19 (3.84, 4.53)	-0.5 (-0.94, -0.06)	0.027	-0.72 (-1.17, -0.27)	0.002	-0.74 (-1.2, -0.29)	0.001
Animal fats	0 – 10	5.38 (5.06, 5.7)	4.83 (4.51, 5.15)	4.47 (4.14, 4.81)	4.44 (4.1, 4.78)	-0.55 (-0.99, -0.11)	0.014	-0.91 (-1.35, -0.46)	<0.001	-0.94 (-1.39, -0.49)	<0.001
Added sugars	0 – 10	0.19 (0.09, 0.29)	0.47 (0.37, 0.57)	0.44 (0.33, 0.55)	0.5 (0.39, 0.61)	0.29 (0.14, 0.43)	<0.001	0.25 (0.11, 0.4)	0.001	0.32 (0.17, 0.46)	<0.001
**Total PHDI-C score**	0 - 150	50.07 (49.14, 51.01)	48.9 (47.95, 49.85)	46.38 (45.39, 47.37)	46.24 (45.24, 47.25)	-1.17 (-2.39, 0.05)	0.06	-3.69 (-4.94, -2.44)	<0.001	-3.83 (-5.09, -2.57)	<0.001

Abbreviations: PHDI-C, Planetary Health Diet Index for children and adolescents; DGV ratio, dark green vegetables ratio; ReV ratio, red and orange vegetables ratio; WC ratio, whole cereals ratio; CI, confidence interval; diff, difference.

[a] Estimates and p-values from mixed effects models adjusting for child and maternal characteristics at baseline, including: child gender (male vs female), child age (3-4 years vs 5-6 years), child weight status (non-overweight, overweight, and obesity), maternal age (<25 years vs ≥25 years), and maternal education (incomplete secondary education, complete secondary education, complete tertiary education), plus dietary recall characteristics including: day of the dietary recall (weekday vs weekend/holiday), type of eating pattern (typical (i.e., recall from a typical day) vs atypical (i.e., recall from a special occasion such as celebrations, sickness or vacations)), and type of diet (normal (i.e., omnivorous diet with no dietary restriction of any kind) vs special diet (e.g., lactose free, gluten free, vegan or vegetarian diets)); n=877 (considers participants who had data collected at baseline (2016) and at least one more wave was performed).

References

1. World Health Organization. *WHO child growth standards. Length/height-for-age, weight-for-age, weight-for-length, weight-for-height and body mass index-forage. Methods and development.*; World Health Organization: Geneva, 2006.
2. de Onis, M.; Onyango, A.W.; Borghi, E.; Siyam, A.; Nishida, C.; Siekmann, J. Development of a WHO growth reference for school-aged children and adolescents. *Bull World Health Organ* **2007**, *85*, 660-667.
3. World Health Organization (WHO). Body mass index - BMI. Available online: http://www.euro.who.int/en/health-topics/disease-prevention/nutrition/a-healthy-lifestyle/body-mass-index-bmi (accessed on
4. Expert Panel on Detection Evaluation and Treatment of High Blood Cholesterol in Adults. Executive Summary of The Third Report of The National Cholesterol Education Program (NCEP) Expert Panel on Detection, Evaluation, And Treatment of High Blood Cholesterol In Adults (Adult Treatment Panel III). *Jama* **2001**, *285*, 2486-2497.
